# Risk factors and preventive interventions for post Covid-19 condition: systematic review

**DOI:** 10.1080/22221751.2022.2140612

**Published:** 2022-11-11

**Authors:** Jennifer Pillay, Sholeh Rahman, Samantha Guitard, Aireen Wingert, Lisa Hartling

**Affiliations:** Alberta Research Centre for Health Evidence (ARCHE), Department of Pediatrics, Faculty of Medicine and Dentistry, University of Alberta, Edmonton, Canada

**Keywords:** Post COVID-19 condition, systematic review, risk factors, prevention, interventions

## Abstract

This systematic review examined pre-existing and clinical risk factors for post Covid-19 condition (≥12 weeks after onset), and interventions during acute and post-acute phases of illness that could potentially prevent post Covid-19 condition. The review focuses on studies collecting data during the early phases of the pandemic and prior to the emergence of variants of concern and widespread vaccination. We searched bibliographic databases and grey literature. Two investigators independently reviewed abstracts and full-text articles, and data extraction and risk of bias assessments were verified. Meta-analysis was performed when suitable and we assessed the certainty of evidence using GRADE. We included 31 studies. We found small-to-moderate associations (e.g. adjusted odds ratios 1.5 to <2.0) between female sex and higher non-recovery, fatigue, and dyspnea (moderate certainty). Severe or critical acute-phase Covid-19 severity (versus not) has probably (moderate certainty) a large association (adjusted ratio ≥2.0) with increased cognitive impairment, a small-to-moderate association with more non-recovery, and a little-to-no association with dyspnea. There may be (low certainty) large associations between hospitalization and increased non-recovery, increased dyspnea, and reduced return to work. Other outcomes had low certainty of small-to-moderate or little-to-no association or very low certainty. Several potential preventive interventions were examined, but effects are very uncertain. Guidelines in relation to surveillance, screening, and other services such as access to sickness and disability benefits, might need to focus on females and those with previously severe Covid-19 illness. Continuous assessment of emerging evidence, especially on whether different variants and vaccination impact outcomes, will be important. PROSPERO registration: CRD42021270354.

## Background

The ongoing global pandemic caused by coronavirus disease 2019 (Covid-19) has affected nearly 270 million people so far and has resulted in more than 5 million deaths worldwide [1]. While most people infected with Covid-19 typically recover within a few weeks [2], some may experience persistent symptoms lasting for several weeks or even months after the initial infection [3,4]. Several terminologies and definitions have been proposed to describe prolonged Covid-19 illness [5–8]. The term “post Covid-19 condition” was established via consensus by the WHO as of 6 October 2021, to refer to new or ongoing symptoms in individuals with a history of probable or confirmed SARS-CoV-2 infection, occurring usually 3 months from the onset of the infection, lasting for at least 2 months following initial recovery that cannot be explained by an alternative diagnosis [9]. The exact frequency and nature of post Covid-19 condition remains largely unknown, partly due to the lack of a consistent definition and ascertainment criteria prior to October 2021 [10]. Emerging evidence indicate varying estimates, ranging from 10% to more than 80% of infected individuals suffering from on-going symptoms for weeks or months after the initial infection, with the most commonly reported symptoms being fatigue, weakness, and breathlessness among others [11–14].

Our understanding of factors predisposing to post Covid-19 condition is limited [10,13]. Evidence indicates that occurrence and intensity of post-Covid symptoms may be influenced by several factors, including age, gender, pre-existing conditions, and the level of care received during initial stages of the disease [4,15–19]. There are also uncertainties around whether the type of the treatment received during the acute phase of Covid-19 influences longer-term outcomes [20].

Many local governments and healthcare systems have already been pushed beyond their limits to cope with the rapid spread of acute Covid-19 infection and its serious implications on healthcare utilization and management of resources. Given the timeline of the pandemic and large number of people infected with Covid-19, it is anticipated that post Covid-19 condition will become the new public health challenge to tackle [10]. There is a clear need for better understanding of this emerging threat and indeed, it has been urged to prioritize research in this area [20–22]. Studying factors that predispose an individual to post Covid-19 condition will help to identify high-risk groups, target potential interventions to those groups, and establish effective patient-care pathways. This, in turn, would ensure evidence-based allocation of resources and a better preparedness of health systems to overcome the challenge. Hence, the objective of these systematic reviews was to identify and synthesize evidence around risk factors of post Covid-19 condition and interventions provided to patients during the acute and post-acute phases of the disease that could potentially prevent post Covid-19 condition. The review focuses on studies collecting data during the early phases of the pandemic and prior to the emergence of variants of concern and of widespread vaccination.

## Methods

### Review approach and key questions (KQs)

We undertook two systematic reviews following a pre-defined, registered protocol (CRD42021270354) [23]. The reviews are reported following Preferred Reporting Items for Systematic Reviews and Meta-analyses [24,25]. During protocol development, a working group comprised of members of our research team, representatives from the Public Health Agency of Canada (PHAC) and the Canadian Agency for Drugs and Technologies in Health (CADTH), and clinical experts was formed to refine the review questions and PICOTS components (population, intervention(s) or exposure(s), comparator(s), outcome(s), timing, setting, and study design). The following KQs were determined to be addressed:

**KQ1:** Among people who have had Covid-19, what are the associations between pre-existing and clinical risk factors and development of post Covid-19 condition?

**KQ2:** Among people in the acute (symptom onset to 4 weeks) or early post-acute phase (4–8 weeks) of Covid-19 what are the effects of interventions to prevent post Covid-19 condition?

### Eligibility criteria

Tables S1 and S2 in the Supplement detail our eligibility criteria for each KQ. For the purpose of this review, we used the WHO definition for post Covid-19 condition, as requiring symptoms persisting ≥12 weeks after a positive Covid-19 test or symptom onset.

The population of interest for KQ1 included people of any age in the general population (with/without previous Covid-19) or those with Covid-19. For KQ2, we included people of any age in the acute (0–4 weeks since a positive test/symptom onset) or the post-acute (4–8 weeks) phase of Covid-19; studies could have ≤20% of participants at 9–12 weeks post-Covid. After the protocol development but before data extraction, it was decided by the working group to exclude studies where a majority of the participants had been admitted to an intensive care unit (ICU) during the acute phase, because of the presumed similarity and overlap with post-ICU syndrome for which there are existing treatment pathways and guidelines.

For KQ1, we were interested in pre-existing risk factors (e.g. demographic variables, BMI, specific chronic conditions affecting a relatively large population and found to have an effect on Covid-19 severity [26], Covid-19 vaccination status, etc.) and clinical risk factors arising during acute phase of Covid-19 (e.g. presence of dyspnea, number of symptoms/symptom severity, need for hospitalization or ICU admission, etc.) [27]. We defined the comparator/control as people without the exposure of interest (e.g. male versus female, people without diabetes, etc.) or with different levels of the exposure (e.g. different age group or BMI category). For KQ2, we included any potentially preventive intervention that started before 8 weeks after a positive Covid-19 test/symptom onset (in ≥80% of study participants). The comparator was usual medical care (e.g. supportive care for acute Covid-19); we included studies with no comparator if there were no other studies with a comparator for the intervention.

The outcomes of interest were selected by a rating approach [28]. Groups of clinical experts, policy makers, and patients were asked to rate the importance of each proposed outcome in terms of how important the effect of a risk factor or intervention on the outcome would be for patients with long-term symptoms, and for the government to offer new types of healthcare services to improve the outcome. The rating was on a 9-point scale, with scores 0–3 indicating “not very important,” 4–6 indicating “important but not critical,” and 7–9 indicating “critical” outcomes. Based on this rating, we included outcomes rated as “critical” for both KQs, which included: non-recovery/persistent symptoms; major cardiovascular event or organ impairment; moderate/severe or persistent (≥3 weeks) fatigue, breathlessness/dyspnea, impairment in functional capacity, cognitive impairment, sleep disturbances, pain including headaches and chest pain; important impact on quality of life; clinical/pathological levels of psychopathology (e.g. anxiety, depression, post-traumatic stress disorder); and unable to return to full-time work/school/education or caring role. For KQ2, due to the expectation of scarce evidence we also included outcomes rated as important, including: all-cause hospital admission, emergency department visits, requiring urgent care outside of hospital, requiring referral for specialist care for physical and/or mental health (may include new onset of disease such as diabetes), requiring pulmonary rehabilitation and/or long-term oxygen therapy, and any or serious adverse effects of the intervention/treatment. For KQ1, we included outcomes assessed at least 12 weeks after Covid-19 diagnosis or symptom onset (including studies where mean follow-up duration ± 1 standard deviation was ≥12 weeks). For KQ2, the post-baseline follow-up had to be at least 3 weeks and outcomes measured ≥12 weeks post-Covid. If outcomes were assessed during follow-up from an intervention and included <12 week data (e.g. hospitalization during follow-up), we looked for data specific to event timing (e.g. in figures or text) or contacted authors for data on events occurring ≥12 weeks post-Covid.

We included peer-reviewed articles, results in trial registrations (if no report published yet), and pre-prints of primary studies, including (for KQ1) prospective/retrospective observational studies with ≥ 300 participants with Covid-19, and (for KQ2) randomized and quasi-randomized or experimental studies (e.g. controlled before-after, interrupted time series, uncontrolled before-after implementation studies), prospective or retrospective cohort studies with control groups, case–control studies, and case series/uncontrolled cohorts. For uncontrolled studies reporting continuous outcomes, baseline and follow-up scores needed to be reported.

For all non-randomized studies, we prioritized multivariable adjusted data (or other similar adjustment methods, i.e. matching/stratification) where at least age, sex (when applicable), some measure of Covid-19 illness severity (e.g. hospitalization, ICU admission, etc.), and comorbidities were taken into account. For KQ1, we included studies with adjustment for at least two of the four variables, whereas for KQ2 we included all studies regardless of adjustment.

### Literature search and study selection

The search strategies for each KQ (Supplement) were developed by a research librarian and peer-reviewed by a second librarian using the PRESS 2015 checklist [29]. Concepts related to post Covid-19 were combined with concepts related to risk factors (KQ1) and interventions (KQ2). Search vocabulary and syntax were adjusted across databases. In order to facilitate search updates, the searches were conducted in Ovid using a multifile search for Medline® including Epub Ahead of Print, In-Process & Other Non-Indexed Citations and Embase. We limited the search to studies in English or French from any country/setting, published from January 2021 (KQ1) and June 2020 (KQ2) onwards. The date limit was applied given the timeline of Covid-19 emergence and our focus on long-term outcomes, and (for KQ1) because we had access to other reviews from which to locate studies published earlier [12,13,30]. Moreover, our earlier review on risk factors associated with Covid-19 severity (including long-term outcomes) with literature search updated in April 2021, did not identify any studies prior to Fall 2020 [26]. These searches were run on 12 August 2021 (KQ1) and 28 July 2021 (KQ2). Additionally, we searched Clinicaltrials.gov and several organizational websites based on previous input on those most relevant to Canada, including the Government of Canada’s First Nations and Inuit Health Branch, PHAC, United States Centers for Disease Control and Prevention, Public Health England, European Centre for Disease Prevention and Control, CADTH Covid-19 Evidence Portal, and the reference lists of the included studies and relevant systematic reviews.

Search results were uploaded to an EndNote library (v. X9, Clarivate Analytics, Philadelphia, PA), duplicates were removed, and then were exported to DistillerSR (https://www.evidencepartners.com/). Following the pre-defined eligibility criteria, a two-step study selection was done in DistillerSR, first by title-abstract (screening) and then by full-text (selection). Before each step, all reviewers involved in study selection piloted a random sample of records to resolve any ambiguousness. During screening, we applied the liberal accelerated method whereby each title-abstract requires one reviewer to include but two to exclude. Any potentially relevant record was retained for full-text review. Study selection was done in duplicate (i.e. two independent reviewers) with arbitration by a third reviewer in case of a disagreement. If additional information was required to make a final decision on a study, we contacted the corresponding author twice via e-mail over two weeks. We excluded the study if there was no response after the two attempts. We documented the screening process in a PRISMA flow-diagram and recorded the reasons for all full-text exclusions.

### Data extraction and management

We developed standardized data extraction forms in Excel. After piloting the form, one reviewer extracted data from the included studies independently and a second reviewer verified all data for accuracy and completeness. Disagreements were resolved by discussion or by consulting a third reviewer.

From each study, we extracted information related to: study characteristics, population characteristics, setting and type of care during acute phase, risk factors (KQ1) or intervention characteristics (KQ2) and comparators, follow-up length, analysis details, outcome definitions and ascertainment, and results data. For all outcomes we prioritized including dichotomous (e.g. proportion with event or important degree of dysfunction) over continuous (e.g. mean score) data, and data attributed to or changed since Covid-19 (e.g. change in outcome pre versus post-Covid). We also prioritized outcomes assessed using a valid measurement tool/scale that best represented the outcome domain [23] and did not include those based on single question about experiencing a symptom unless it indicated the symptoms were persistent/moderate/severe. In cases of missing or unclear information, we contacted the study authors for clarification, and ceased contact after two attempts if we received no response. In the case of multiple analyses reported for an outcome in observational studies, we extracted the most adjusted data. If data for outcomes at multiple time points were reported we selected the data closest to 12 and 24 months, with a preference for longer versus shorter time. We converted continuous to binary data where possible, to enable a pooled estimate across several studies [23].

### Risk of bias assessment

We assessed risk of bias of included studies using the JBI critical appraisal checklist for cohort studies [31] (KQ1), Cochrane RoB 2.0 for randomized studies [32] and JBI critical appraisal checklist (quasi-experimental) for quasi-randomized and other experimental studies, such as controlled before-after studies [31] (KQ2). For all study designs, we added a question about Covid-19 ascertainment (domain considered as low risk if ≥90% were lab-confirmed). For KQ1, we collected information about missing outcome data (i.e. measured as per methods but no results reported), and for KQ2 observational studies, we added three additional questions about selective reporting, missing outcome data, and whether a sufficient amount of eligible patients (>50%) were enrolled. The overall risk of bias for an outcome was considered “low” if all domains were at low risk of bias, of “some concern” if fewer than two domains were at high risk of bias and we did not feel the study conclusions would be impacted by that domain (e.g. ascertainment of exposure), and “high” if ≥2 domains were assessed as being high risk.

After piloting each tool on a sample of studies, one reviewer assessed each study independently, followed by verification by a second reviewer. Disagreements were resolved by discussion or arbitration by a third reviewer, if needed. To inform our assessment, we used information from all publications associated with any study as well as trial registrations and protocols for KQ2 studies. We did not exclude studies due to high risk of bias, however, risk of bias was considered when assessing the certainty of evidence using the Grading of Recommendations, Assessment, Development and Evaluation approach (GRADE).

### Data synthesis

For both KQs, we charted the study data in terms of the main variables of intervention/exposure, Covid-19 illness severity (hospitalized, mixed, non-hospitalized), timing of outcome measurement, and outcome. As pre-specified, we considered a meta-analysis if there was enough clinical and methodological similarity in an exposure-outcome comparison and data for that comparison was available in at least 75% of the studies. For the risk factors related to acute-phase illness severity (e.g. critical/severe illness vs. not), with the exception of need for hospitalization which was only analysed in mixed populations, we analysed data separately for hospitalized, mixed severity and non-hospitalized populations to avoid confounding. Although acute Covid-19 severity was defined using different standards across the studies, we pooled estimates based on similarities in the description of each level of severity (e.g. requiring mechanical/non-mechanical ventilation, high flow oxygenation, etc.). Because of the timing of data collection in the included studies, the large majority of participants were exposed during the early phase of the pandemic and were not vaccinated, such that variants of concern and vaccination status were not important potential confounders in this review. Analysis was performed in Review Manager (RevMan, v.5.3, Copenhagen: The Nordic Cochrane Centre, the Cochrane Collaboration, 2014). For KQ1 and studies with a control group in KQ2, we planned to use a pairwise random-effects meta-analysis. We employed a generic inverse variance method for KQ1 because of the preference for using relative measures from adjusted analysis. For all syntheses whether or not from meta-analysis, we categorized findings by magnitude: a relative effect/association of 0.75–1.49 was considered as “little-to-no,” whereas 0.50–0.74 and 1.5–1.99 were “small-to-moderate” and <0.50 or ≥2.00 were “large” effects/associations for fewer/benefit or more/harm, respectively. In absence of a meta-analysis, consensus was made on a best estimate (within one of these categories) of effect/association across studies while considering their relative weight by sample size. As pre-defined, we considered several variables for either grouping studies or conducting subgroup analyses if substantial heterogeneity existed in magnitude (e.g. different categories of conclusions) or direction of association/effects, including hospitalized versus non-hospitalized/mixed population, lab-confirmed Covid-19 versus otherwise, timing of outcome assessment (e.g. 12–22 weeks versus ≥22 weeks), symptom severity for both KQs as well as components of the intervention for KQ2 (e.g. enrolment in acute versus post-acute phase, online versus in-person intervention, follow-up timing). We also conducted sensitivity analysis by removing studies having high risk of bias, particularly those in KQ1 that only sufficiently reported univariate analyses despite conducting multivariate analysis. We would have tested for small study effects using funnel plots and Egger’s regression test if an analysis included at least 10 studies.

### Certainty of evidence

The certainty of evidence for each exposure-outcome association across the studies was assessed by at least two reviewers using GRADE [33,34]. We assessed our certainty in the categorical conclusions about the magnitude of association/effect as described above, though if an association had higher certainty at a smaller magnitude (small-to-moderate vs. large association) we chose to report the higher certainty magnitude. For observational studies, we started at high certainty for KQ1 [35] and low certainty for KQ2 [28]. For randomized and non-randomized trials in KQ2, we started at high certainty. We rated down the certainty for concerns related to risk of bias, indirectness (mainly in terms of whether reported outcome measures were a good conceptual match to our outcomes of interest), inconsistency (in direction and/or magnitude of effect) across the studies or lack of consistency (single studies), imprecision (95% confidence intervals indicating the effect/association may allow for more than one conclusion e.g. little-to-no and a small-to-moderate association), and/or reporting bias domains by one or two levels depending on how serious the concerns were, that is how much overall conclusions appear to be impacted by the domain. The final certainty of evidence (i.e. high, moderate, low, very low) and the reasoning for each are presented in summary tables.

## Results

### KQ1: risk factors

**Figure 1.**
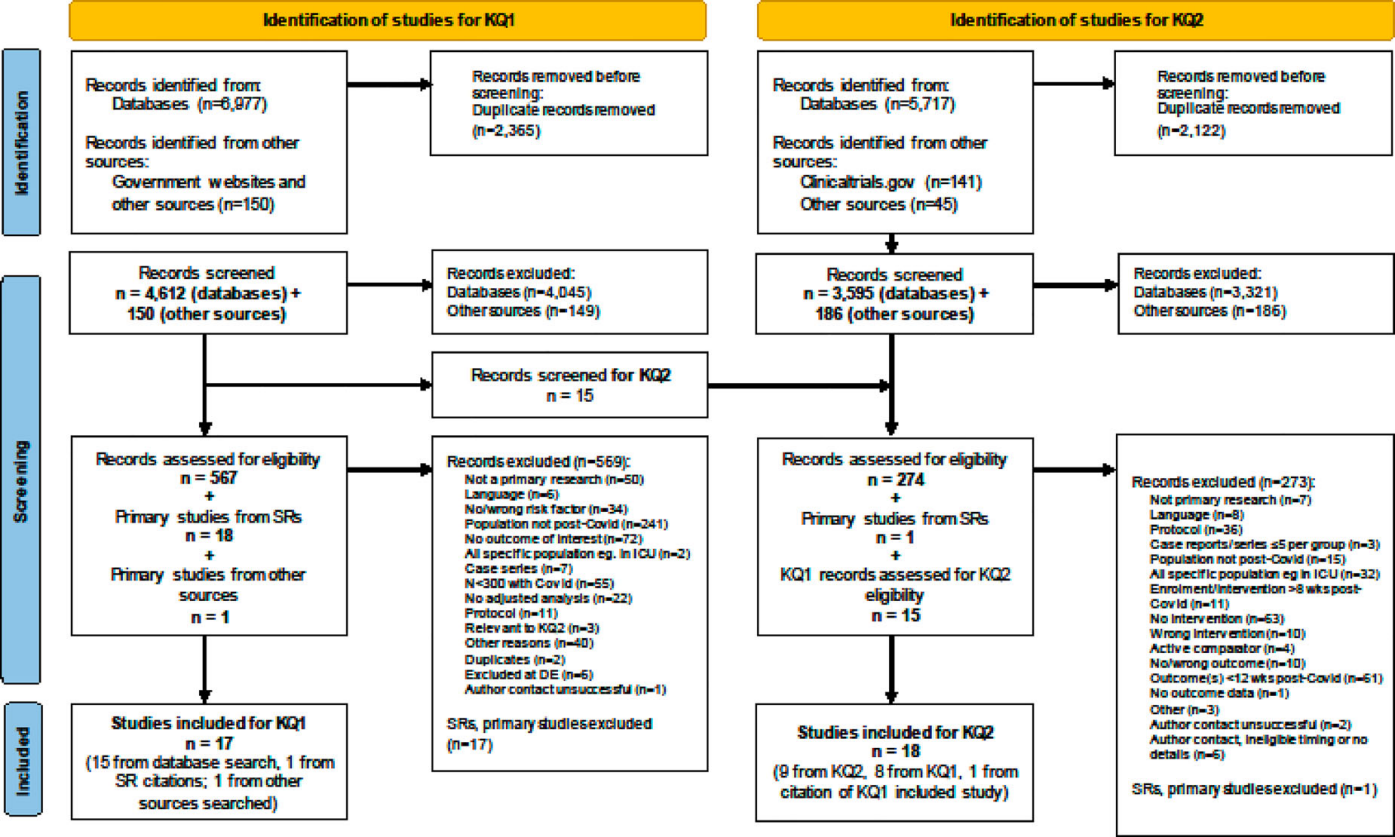
Flow of literature.

We identified 4612 records from searching databases and 150 records from other sources; 17 unique records met the eligibility criteria and were included (Figure 1; see Supplement for the lists of excluded studies for KQs 1 and 2) [4,14–19,36–45]. Study characteristics are included in Table 1 and Table S3. The studies originated from China (*n* = 5), Italy (*n* = 2), Norway (*n* = 2), Russia (*n* = 2), Switzerland (*n* = 2), and one each from the UK, USA, Sweden, and Turkey. Fourteen studies were classified as prospective and three as retrospective cohort studies. The studies included a median of 540 participants (range: 304–11,955), with lab-confirmed Covid-19 in majority of studies (*n* = 12), and varying baseline Covid-19 severity (hospitalized *n* = 9, non-hospitalized *n* = 3, mixed severity *n* = 5). Of the included studies, only one was exclusively in children [40]. The median age in other studies was 53.0 years (range: 42.7–69.0) and the median male proportion across the studies was 49%. Most of the studies (*n* = 12) were assessed as having some concern for risk of bias, mainly due to issues arising from incomplete follow-up data, insufficient statistical adjustment (e.g. results not adjusted for comorbidities or illness severity), or outcome/exposure assessment method (i.e. not measured with valid tools) (Table S4). Only one study [37] was assessed as having low risk of bias, which included elderly people previously hospitalized with Covid-19.

**Table 1.** Summary of characteristics of studies included in key questions 1 and 2.

Characteristics	KQ1 studies (*n* = 17)	KQ2 studies (*n* = 18)
Study design (*n*)	Prospective controlled cohort (14) Retrospective controlled cohort (3)	Pilot RCT(1) Non-randomized experimental design (2) Prospective controlled cohort (2) Prospective uncontrolled cohort (4) Retrospective controlled cohort^a^(9)
Country (*n*)	China (5) Italy (2) Norway (2) Russia (2) Switzerland (2) USA (1) UK (1) Sweden (1) Turkey (1)	China (7) USA (3) Italy (2) India (1) Iran (1) Belgium (1) Norway (1) Spain (1) Switzerland (1)
Population	Sample size, median (range): 540 (304–11,955) Age, median (range)^b^: 53.0 (42.7–69.0) Male %, median (range): 49.0 (32.9–65.0)	Sample size, median (range): 82 (10–538) Age, median (range): 57.0 (34.2–70.0) Male %: median (range): 56.5 (36.0–100.0)
Covid-19 ascertainment (*n*)	≥90% lab-confirmed (12) <90% lab confirmed (4) NR (1)	≥90% lab confirmed (12) <90% lab confirmed (1) NR (5)
Baseline Covid-19 severity (*n*)	Hospitalized (9) Mixed severity^c^(5) Non-hospitalized (3)	Hospitalized (17) Mixed severity (1)
Outcome assessment timing (*n*)	12–21 weeks (3) ≥22 weeks (14)	12–16 weeks (12) >16 weeks (6)
Risk of bias (*n*)	Some concerns (11) High (5) Low (1)	Some concerns (6) High (12) Low (0)

Abbreviations: NR: not reported; RCT: randomized controlled trial.

^a^ These studies were prospective for the authors’ main objective but retrospective for the purposes of this review which analysed data on treatment(s) received during acute illness.

^b^ Excluding the one study that included children (Osmanov et al.) [40].

^c^ Studies including hospitalized and non-hospitalized participants.

The certainty of evidence was downgraded for all risk factor-outcome associations, mostly due to concerns related to risk of bias, inconsistency (i.e. single study or inconsistent findings across studies), and/or indirectness (e.g. reported outcomes not aligning with review question). Table 2 summarizes the findings where the evidence was of low or greater certainty; Tables S5–S8 contain more detail including individual study and meta-analysis results, including risk factors for which there was very low certainty.

**Table 2.** Summary of evidence for associations between risk factors and post Covid-19 condition with moderate or low level of certainty.

Variable	Outcome and timing	Baseline severity (no. of studies)	Conclusion*	Certainty^†^
Demographic risk factors				
Age (continuous)	Non-recovery in children			
Age group (ref: <2 yrs)	≥22 weeks	Hospitalized (*n* = 1)	Small-to-moderate association with more post-Covid condition among children ≥6 vs. <2 years old	Low^a,c^
Age (continuous)	Non-recovery			
	12–21 weeks	Hospitalized (*n* = 1)	Little-to-no association	Low^a,b^
	≥22 weeks	Hospitalized (*n* = 1)		
Age group (18–40 [ref]; 40–60; >60 years)	≥22 weeks	Hospitalized (*n* = 2)	Little-to-no association	Low^a,c^
		Mixed (*n* = 2)		
		Non-hospitalized (*n* = 2)		
Sex (ref: Male)	12–21 weeks	Hospitalized (*n* = 1)	Small-to-moderate association with more post-Covid condition among females	Moderate^a^
	≥22 weeks	Hospitalized (*n* = 3)		
		Mixed (*n* = 2)		
		Non-hospitalized (*n* = 2)		
Age (continuous)	Fatigue			
	12–21 weeks	Non-hospitalized (*n* = 1)	Little-to-no association	Low^a,b^
	≥22 weeks	Hospitalized (*n* = 2)		
		Mixed (*n* = 1)		
Sex (ref: Male)	12–21 weeks	Non-hospitalized (*n* = 1)	Small-to-moderate association with more fatigue among females	Moderate^a^
	≥22 weeks	Hospitalized (*n* = 4)		
		Mixed (*n* = 2)		
		Non-hospitalized (*n* = 1)		
BMI (continuous)	12–21 weeks	Non-hospitalized (*n* = 1)	Little-to-no association	Low^a,b^
	≥22 weeks	Mixed (*n* = 2)		
Sex (ref: Male)	Dyspnea			
	≥22 weeks	Hospitalized (*n* = 2)	Small-to-moderate association with more dyspnea among females	Moderate^a^
		Mixed (*n* = 1)		
		Non-hospitalized (*n* = 1)		
Age (continuous)	Depression			
	≥22 weeks	Hospitalized (*n* = 2)	Little-to-no association	Low^a,b^
Age group (18–39; 40–64; ≥65 years)	≥22 weeks	Mixed (*n* = 1)	Little-to-no association	Low^a,c^
Age group (≥60 vs. <60 years)	Functional incapacity			
	12–21 weeks	Hospitalized (*n* = 2)	Small-to-moderate association with more incapacity in ≥60 vs. <60 years old	Low^a,b^
Sex (ref: Male)	12–21 weeks	Hospitalized (*n* = 1)	Small-to-moderate association with more functional incapacity among females	Low^a,d^
	≥22 weeks	Hospitalized (*n* = 1)		
Sex (ref: Male)	Cognitive impairment			
	≥22 weeks	Hospitalized (*n* = 2)	Little-to-no association	Low^A^
Race/ethnicity	Return to work			
	≥22 weeks	Hospitalized (*n* = 1)	Small-to-moderate association with less return to work in non-White vs. White people	Low^a,c^
Pre-existing conditions				
Number of comorbidities (≥1 vs. 0)	Non-recovery			
	12–21 weeks	Mixed (*n* = 1)	Small-to-moderate association with more post-Covid condition among people with ≥1 vs. 0 comorbidities	Moderate^a^
	≥22 weeks	Hospitalized (*n* = 1)		
		Mixed (*n* = 1)		
		Non-hospitalized (*n* = 1)		
Diabetes (any)	≥22 weeks	Hospitalized (*n* = 1)	Little-to-no association	Low^a,c^
Chronic cardiac disease	≥22 weeks	Hospitalized (*n* = 1)	Little-to-no association	Low^a,c^
Hypertension	≥22 weeks	Hospitalized (*n* = 1)	Little-to-no association	Low^a,c^
Chronic pulmonary disease	Fatigue			
	≥22 weeks	Hospitalized (*n* = 1)	Small-to-moderate increase with more fatigue among people with chronic pulmonary disease	Low^a,b^
		Mixed (*n* = 1)		
Hypertension	≥22 weeks	Hospitalized (*n* = 1)	Little-to-no association	Low^a,b^
		Mixed (*n* = 1)		
Rheumatological disorder	≥22 weeks	Hospitalized (*n* = 1)	Little-to-no association	Low^a,b^
		Mixed (*n* = 1)		
Chronic pulmonary disease	Dyspnea			
	≥22 weeks	Hospitalized (*n* = 1)	Little-to-no association	Low^a,d^
		Mixed (*n* = 1)		
Hypertension	≥22 weeks	Hospitalized (*n* = 1)	Little-to-no association	Low^a,c^
Number of comorbidities (≥1 vs. none)	Psychopathology (depression/anxiety)			
	≥22 weeks	Hospitalized (*n* = 1)	Little-to-no association	Low^a,d^
		Mixed (*n* = 1)		
Rheumatologic disorder	≥22 weeks	Hospitalized (*n* = 1)	Small-to-moderate association with more psychopathology among people with rheumatologic disorders	Low^a,c^
COPD	Cognitive impairment			
	≥22 weeks	Hospitalization (*n* = 1)	Small-to-moderate association with more cognitive impairment among people with COPD	Low^a,c^
Hypertension	≥22 weeks	Hospitalization (*n* = 1)	Little-to-no association (among older adults)	Low^a,c^
Acute phase Covid-19 illness severity				
Acute Covid-19 severity (severe/critical vs. not)	Non-recovery			
	12–21 weeks	Mixed (*n* = 1)	Small-to-moderate association with more post-Covid condition among people who had severe/critical Covid-19 illness severity in the acute phase	Moderate^a^
	≥22 weeks	Mixed (*n* = 1)		
	≥22 weeks	Hospitalized (*n* = 3)	Little-to-no association	Low^a,c^
No. of symptoms (≥1 vs. 0)	≥22 weeks	Mixed (*n* = 1)	Small-to-moderate association with more post-Covid condition among people who had ≥1 vs. 0 Covid-19 symptoms in the acute phase	Low^a,c^
No. of symptoms (ref: ≤ 2 symptoms)	≥22 weeks	Non-hospitalized (*n* = 1)	Small-to-moderate association with more post-Covid condition among people who had >2 vs. ≤2 symptoms in the acute phase	Low^a,c^
Need for hospitalization	12–21 weeks	Mixed (*n* = 1)	Large association with more post-Covid condition at 12–21 weeks among people who were hospitalized in the acute phase	Low^a,c^
	≥22 weeks	Mixed (*n* = 2)	Small-to-moderate association with more post-Covid condition at ≥22 weeks among people who were hospitalized in the acute phase	Low^a,d^
No. of symptoms (ref: 0–5 symptoms)	Fatigue			
	12–21 weeks	Non-hospitalized (*n* = 1)	Small-to-moderate association with more fatigue among people who had >5 vs. ≤5 symptoms in the acute phase	Low^a,c^
Acute Covid-19 severity (severe/critical vs. not)	Dyspnea			
	≥22 weeks	Hospitalized (*n* = 2)	Little-to-no association	Moderate^a^
Need for hospitalization	≥22 weeks	Mixed (*n* = 1)	Large association with more dyspnea among people who were hospitalized in the acute phase	Low^a,c^
Acute Covid-19 severity (severe/critical vs. not)	Depression			
	≥22 weeks	Hospitalized (*n* = 2)	Small-to-moderate with more psychopathology (depression) among people who had severe/critical Covid-19 illness severity in the acute phase	Low^a,d^
Need for intubation	Functional incapacity			
	≥22 weeks	Hospitalized (*n* = 1)	Small-to-moderate association with more functional incapacity among people requiring intubation in the acute phase	Low^a,c^
Acute Covid-19 severity (severe/critical vs. no)	Cognitive impairment			
	≥22 weeks	Hospitalized (*n* = 2)	Large association with more cognitive impairment among people who had severe/critical Covid-19 illness severity in the acute phase	Moderate^a^
Need for hospitalization	Return to work			
	≥22 weeks	Mixed (*n* = 1)	Large association with less return to work among people who required hospitalization in the acute phase	Low^a,c^

Abbreviations: CI: confidence interval; Ref: reference; vs: versus.

*Our conclusions were based on applying thresholds of association. A relative effect (OR/RR) of 0.75–1.49 was considered as little-to-no association, 0.50–0.74 (decrease) and 1.5–1.99 (increase) as small-to-moderate, and <0.50 (decrease) or ≥2.00 (increase) as large effect/association.

^†^ Assessed using Grading of Recommendations, Assessment, Development and Evaluation. We rated the certainty based on a pooled estimate in case of a meta-analysis or a best estimate of effect/association (relative to the thresholds) across studies based on individual study results considering their sample size and measures of variance in absence of a meta-analysis. Certainty started at high and was rated down by 0, 1, or 2 levels for risk of bias (a), indirectness in outcome (b), inconsistency/lack of consistency (c), and imprecision/ wide confidence intervals (d); capital letters indicate very serious concern.

#### Demographic risk factors

The certainty of evidence was moderate for a small-to-moderate association between female sex and higher: non-recovery (8 studies, *n* = 6613) [15–18,38,39,41,43], fatigue (8 studies, *n* = 7116) [14,15,17,36,38,39,43,44], and dyspnea (4 studies, *n* = 3817) [15,17,38,39]. The certainty of evidence was low for a small-to-moderate association with higher: non-recovery among children aged ≥6 versus <2 years (1 study, *n* = 518) [40], functional incapacity in hospitalized adults aged ≥60 versus <60 years old (2 studies, *n* = 867) [15,42], functional incapacity in females (2 studies, *n* = 867) [15,42], and lower return to work (at a median of 6.7 months from symptom onset) in non-White people (1 study, *n* = 382) [19]. Several findings had low certainty for little-to-no association, and several risk factors had very low certainty evidence (Table S5).

#### Pre-existing conditions

The certainty of evidence was low for a small-to-moderate association between: number of comorbidities (i.e. ≥ 1 versus 0) and non-recovery (4 studies, *n* = 2069) [4,15–17], chronic pulmonary disease with fatigue (2 studies, *n* = 2961) [36,38], rheumatologic disorder with depression/anxiety (1 study, *n* = 2649) [38], and chronic obstructive pulmonary disease (COPD) or hypertension with cognitive impairment (1 study, *n* = 1539) [37]. Post Covid-19 condition was found to have little-to-no association (low certainty) with a few other pre-existing conditions, including diabetes and cardiovascular diseases, and there was very low certainty for several other pre-existing conditions (Table S6).

#### Acute-phase Covid-19 illness severity

Severe or critical acute Covid-19 illness severity (versus not) showed a moderate certainty of evidence for a large association with cognitive impairment (hospitalized populations; 2 studies, *n* = 2335) [37,43], small-to-moderate association with non-recovery (mixed-severity populations; 2 studies, *n* = 1438) [4,17], and little-to-no association with dyspnea (hospitalized populations; 2 studies, *n* = 2976) [15,38]. There was a low certainty of evidence for a large association between hospitalization during the acute phase with: non-recovery (1 study, *n* = 1007) [4], more dyspnea (1 study, *n* = 431) [17], and reduced return to work (1 study, *n* = 11,955) [45].

The certainty of evidence was low for a small-to-moderate association between ≥1 versus 0 acute Covid-19 symptoms (1 study, *n* = 599) [18] and ≥3 versus ≤2 symptoms (1 study, *n* = 304) [16] with non-recovery. A small-to-moderate association with low certainty was also found between: number of acute Covid-19 symptoms and fatigue (1 study, *n* = 458) [44], severe or critical acute Covid-19 severity (versus not) and depression (2 studies, *n* = 4382) [14,38], and need for intubation and functional incapacity (1 study, *n* = 382) [19]. Table S7 includes detailed findings by study and synthesis.

### KQ2: preventive interventions

We identified 3595 records from searching databases and 186 records from other sources; 18 unique records, including 8 studies identified through the KQ1 search, met the eligibility criteria and were included (Figure 1; Table 1 and Table S9) [14,19,36,41,46–59]. Several of the studies originated from China (*n* = 7); others were from USA (*n* = 3), Italy (*n* = 2), and one each from India, Iran, Belgium, Norway, Spain and Switzerland. Nine studies were classified as retrospective controlled cohorts; others were prospective (controlled/uncontrolled) cohorts (*n* = 6), non-randomized experimental (*n* = 2) and a pilot randomized controlled trial [56]. The studies included a median of 82 participants (range: 10–538) with 100% lab-conformed Covid-19 in majority of studies (*n* = 14). Only one study involved hospitalized and home-isolated participants [36], while the other 17 studies were among hospitalized individuals with mixed severity (ranging from being asymptomatic to severe/critical). We assessed the risk of bias for objective (i.e. measured) and subjective (i.e. self-reported) outcomes separately; none of the studies were rated as having low risk of bias across all outcomes (Tables S10–S12). The only randomized study identified was a pilot trial with 10 participants [56] that had concerns in several domains related to randomization, blinding, outcome measurement, and reporting the findings. The most common sources of potential bias in other non-randomized studies (experimental and cohort) were related to unreliable outcome measurement, incomplete follow-up, and not accounting for confounding factors.

Interventions identified as potentially preventing post Covid-19 condition included standard medication [14,19,36,41,47,50,56–59], traditional/ayurvedic medication [52,53], stem cell therapy [49], rehabilitation or similar therapies [46,51,54], and screening and referrals [48,55]. We grouped interventions by timing, as those implemented during acute (symptom onset to 4 weeks) phase of Covid-19 with outcomes assessed at shorter (12–16 weeks) or longer (>16 weeks) follow-up duration post Covid-19; and interventions implemented during early post-acute (4–8 weeks) phase of Covid-19 with aforementioned shorter and longer follow-up time (Table 3).

**Table 3.** Summary of evidence for preventive interventions for post Covid-19 condition, by timing of implementation and follow-up duration.

Author, country; study design	Intervention/exposure and comparator with sample sizes	Acute phase treatment; Covid-19 illness severity; ≥90% Laboratory confirmed (Y/N)	Outcomes and results	Conclusions (certainty*) Apply to each outcome seperately
Acute-phase interventions with follow-up 12–16 weeks post-Covid 19				
**Anastasio 2021**, Italy Retrospective controlled cohort	**Steroid use**(*n* = 42) vs. no steroid use (*n* = 180)	Hospitalized 100% pneumonia (in analysis) Y	**Dyspnea (MMRC scale)**: positively correlated *p* = .05	Very uncertain about effects (Very low^a,c^)
**Qin 2021**, China Retrospective controlled cohort	**Corticosteroid use**(*n* = 17) vs. no corticosteroid use (*n* = 64)	Hospitalized Moderate-to-critical Y	**Dyspnea**: DLCO <80% predicted: IG: 10 (59%) vs. CG: 34 (53%); OR: 1.3 (0.4–3.7), *p* = .68	
**Xiong 2021**, China Retrospective controlled cohort	**Corticosteroid use**(*n* = 138) vs. no corticosteroid use (*n* = 400)	Hospitalized Moderate-to-critical Y	**Fatigue**(questionnaire item): IG: 39 (28%) vs. CG: 113 (28%) **Dyspnea**(post-activity polypnoea item): IG: 34 (25%) vs. CG: 81 (20%); RR 1.29 (0.81–2.03)	
**Zhao 2020**, China Retrospective controlled cohort	**Low-dose corticosteroid use**(*n* = 7) vs. no corticosteroid use (*n* = 48)	Hospitalized Moderate-to-severe Y	**Dyspnea**: DLCO <80% predicted: IG: 1 (14.3%) vs. CG: 8 (16.7%); RR 0.83 (0.09–7.90)	
**Gherlone 2021**, Italy Retrospective controlled cohort	**Antibiotic use**(*n* = 102) vs. no antibiotic use (*n* = 20)	Hospitalized Moderate-to-critical (25% ICU) Y	**Adverse events**: association between antibiotic use and salivary gland ectasia: aOR 8.34 (95% CI, 1.47–158.19); *p* = .049	Very uncertain about effects (Very low^a,b,c,D^)
**Kataria 2021**, India Nonrandomized experimental study (nonconcurrent groups)	**Ayurvedic formulation***Tinospora cordifolia*(Guduchi) and *Piper longum* (Pippali) twice daily (*n* = 30) vs. standard care only (*n* = 30)	Hospitalized Asymptomatic (32%) to moderate (3%) Y	**Quality of Life**(general health much better now): IG 18 (64.3%) vs. 15 (51.7%): RR, 1.24 [0.79, 1.94] **Dyspnea**(breathlessness during physical activities): IG 6 (21.4%) vs. CG 3 (10.3%); RR, 2.00 [0.55, 7.22] **Functional incapacity**(work efficiency limited a lot since discharge): IG 0 vs. CG 0 **Fatigue**: 1 (5%) vs. CG 6 (26.1%); RR, 0.17 [0.02, 1.30] **Sleep disturbances**: IG 3 (10.7%) vs. CG 1 (3.4%); RR, 3.00 [0.33, 27.12]	Very uncertain about effects (Very low^a,b,c,d^)
			**Pain** Frequent headaches: IG 3 (10.7%) vs. CG 3 (10.3%); RR, 1.00 [0.22, 4.54] Chest pain: IG 1 (5%) vs. CG 4 (17.4%); RR, 0.25 [0.03, 2.10] **Dyspnea**(oxygen support at home): IG 0 vs. CG 0	Very uncertain about effects (Very low^a,c,d^)
**Feng 2021**, China Nonrandomized experimental study	**Human umbilical cord mesenchymal stem cells**; 1 intravenous delivery (*n* = 12) vs. standard medical care only (*n* = 29)	Hospitalized Severe Y	**Quality of Life**: IG 15.3 ± 3.7 vs. CG 31.9 ± 8.8; MD, −16.60 [−21.23, −11.97] **Dyspnea** Wheezing: IG 3 (37.5%) vs. 15 (75%); RR, 0.50 [0.20, 1.27] Lung function: FEV1/ FVC ratio <70% IG 1 (12.5%) vs. CG 13 (65%); RR, 0.19 [0.03, 1.24] **Adverse events**: 0 **Serious adverse events**: 0	Very uncertain about effects (Very low^a,c,D^)
			**Fatigue**: IG 4 (50%) vs. CG 14 (70%); RR, 0.71 [0.34, 1.51]	Very uncertain about effects (Very low^a,b,c,D^)
**Jain 2021**, U.S. Prospective uncontrolled cohort	**Regional****in-patient rehabilitation** following acute hospitalization	Hospitalized Severe (% ICU NR; 94% required oxygen) NR	**Hospital re-admissions**(within last 2–3 weeks of follow-up): 0	Very uncertain about effects (Very low^a,c,D^)
Acute-phase interventions with follow-up >16 weeks post-Covid 19				
**Blomberg 2021**, Norway Retrospective controlled cohort	**Antibiotic use**(*n* = 31) vs. no antibiotic use (*n* = 262)	Hospitalized or home isolated In hospitalized: 9% on respirator Y	**Fatigue**(Chalder fatigue scale (score ≥4)): IG 17 (55%) vs. CG 91 (35%); aOR 0.42 (0.10–1.75), *p* > .05	Very uncertain about effects (Very low^a,c^)
**Frontera, 2021**, US Retrospective controlled cohort	**Azithromycin use**(*n* = 246) vs. no azithromycin use (*n* = 136)	Hospitalized 8.3% ICU Y	All *p* > .10, unadjusted **Functional incapacity**: (mRS 3–6): OR 0.398 **Functional incapacity**(Barthel Index <100): OR 0.078 **Cognitive impairment**(Telephone Montreal Cognitive Assessment [score < 18]): OR 0.188 **Anxiety**(subscale of Neuro-QoL [T-score > 50]): OR 0.606 **Depression**(subscale of Neuro-QoL [T-score > 50]): OR 0.104 **Fatigue (**subscale of Neuro-QoL [T-score > 50]): OR 0.633 **Sleep disturbances** (subscale of Neuro-QoL [T-score > 50]): OR 0.819 **Return to work**: OR 0.458	
**Frontera, 2021**, US Retrospective controlled cohort	**Corticosteroid use**(*n* = 101) vs. no corticosteroids (*n* = 281)	Hospitalized 32% ICU Y	Unless stated, all *p* > .10 unadjusted; all non-significant with multivariate (data not shown) **Functional incapacity**: (mRS 3–6): OR 0.13 **Functional incapacity**(Barthel Index <100): OR 0.480 (0.29–0.80), *p* = .005 **Cognitive impairment**(Telephone Montreal Cognitive Assessment [score < 18]): OR 0.98 **Anxiety**(subscale of Neuro-QoL [T-score > 50]): OR 0.82 **Depression**(subscale of Neuro-QoL [T-score > 50]): OR 0.37 **Fatigue** (subscale of Neuro-QoL [T-score > 50]): OR 2.2 (1.3–3.9), *p* = .004 **Sleep disturbances** (subscale of Neuro-QoL [T-score > 50]): OR 1.7 (1.0–2.8), *p* = .064 **Return to work** (self-report, Yes/No): OR 0.4 (0.2–0.8), *p* = .008	Very uncertain about effects (Very low^a,c^)
**Huang 2021**, China Retrospective controlled cohort	**Corticosteroid use**(*n* = 398) vs. no corticosteroid use (*n* = 1335)	Hospitalized 4% ICU Y	**Dyspnea**: (Lung function): aOR 1.18 (0.60–2.34), *p* = .63	
			**Anxiety or depression**: aOR 1.23 (0.88–1.72), *p* = .22 **Fatigue or muscle weakness**: aOR 1.04 (0.77–1.42), *p* = .78	
**Frontera 2021**, US Retrospective controlled cohort	**Hydroxychloroquine use**(*n* = 272) vs. no hydroxychloroquine use (*n* = 110) **Therapeutic anticoagulation use**(*n* = 134) vs. no therapeutic anticoagulation use (*n* = 248) **Zinc use**(*n* = NR) vs. no zinc use (*n* = NR)	Hospitalized 32.2% ICU, 51.3% neurological COVID-19 disorder Y	**Functional incapacity**: (mRS 3–6): Hydroxychloroquine: OR 0.98 Therapeutic anticoagulation: OR 0.18 Zinc: OR 0.67 (0.44–1.03), *p* = .066 **Functional capacity**(Barthel Index <100): Hydroxychloroquine: OR 0.97 Therapeutic anticoagulation: OR 0.078 Zinc: OR 0.41 **Cognitive impairment**(Telephone Montreal Cognitive Assessment [score < 18]): Hydroxychloroquine: OR 0.13 Therapeutic anticoagulation: OR 0.99 Zinc: OR 0.52 **Anxiety**(subscale of Neuro-QoL [T-score > 50]): Hydroxychloroquine: OR 0.95 Therapeutic anticoagulation: OR 0.91 Zinc: OR 0.91 **Depression**(subscale of Neuro-QoL [T-score > 50]): Hydroxychloroquine: OR 0.50 Therapeutic anticoagulation: OR 0.98 Zinc: OR 0.62 **Fatigue** (subscale of Neuro-QoL [T-score > 50]): Hydroxychloroquine: OR 0.22 Therapeutic anticoagulation: OR 1.8 (1.1–3.1), *p* = .022 Zinc: OR 0.27 **Sleep disturbances** (subscale of Neuro-QoL [T-score > 50]): Hydroxychloroquine: OR 0.52 Therapeutic anticoagulation: OR 1.7 (1.0–2.8), *p* = .051 Zinc: OR 0.17 **Return to work** (self-report, Yes/No): Hydroxychloroquine: OR 0.19 Therapeutic anticoagulation: OR 0.31 (0.16–0.60), *p* = .001 Zinc: OR 2.3 (1.2–4.4), *p* = .016	Very uncertain about effects (Very low^a,c^)
**Huang 2021**, China Retrospective controlled cohort	**Intravenous immunoglobulin use**(*n* = 345) vs. no intravenous immunoglobulin use (*n* = 1388)	Hospitalized 4% ICU Y	**Dyspnea**(Lung function): aOR 0.94 (0.49–1.79), *p* = .85	Very uncertain about effects (Very low^a,c,d^)
			**Anxiety or depression**: aOR 0.77 (0.54–1.10), *p* = .15 **Fatigue or muscle weakness**: aOR 0.96 (0.70–1.31), *p* = .78	Very uncertain about effects (Very low^a,b^)
**Vetrici 2021**, US Pilot RCT	**Photobiomodulation** adjunctive anti-inflammatory treatment; daily × 4 days (*n* = 5) vs. standard (supportive) care only (*n* = 5)	Hospitalized Moderate/severe (0% IV at baseline) Y	**Dyspnea**(any respiratory symptoms): 0/5 (0%) vs. 4/5 (80%); RR, 0.11 [0.01, 1.64] **Treatment-related adverse events**: 0/5 vs. NA	Very uncertain about effects (Very low^a,b,c,D^)
**Wu 2021**, China Prospective controlled cohort	**Oseltamivir use**(*n* = 53) vs. no oseltamivir use (*n* = 30) **Ganciclovir use**(*n* = 42) vs. no ganciclovir use (*n* = 41)	Hospitalized Severe illness (0% IV) Y	**Dyspnea**: DLCO <80% predicted association with Oseltamivir: unadjusted OR 0.75 (95% CI 0.29–1.92) DLCO <80% predicted association with Ganciclovir: unadjusted OR 1.34 (95% CI 0.53–3.38)	Very uncertain about effects (Very low^a,c,d^)
Early post-acute interventions with follow-up 12–16 weeks post-Covid 19				
**Amini 2021**, Iran Prospective uncontrolled cohort	**Cognitive-motor-therapy**for 4 weeks; performed twice per week; measured at baseline (*n* = 42) and follow-up (*n* = 42)	Hospitalized Non-severe (COVID-19 symptoms stage 1) NR	**Quality of Life**: IG (baseline) 47.8 ± 1.6 vs. IG (3 mo) 46.9 ± 1.2; MD 0.86 (*p* = .006) (<22 pathological symptoms) **Cognitive impairment**: IG (baseline) 17.9 ± 3.3 vs. IG (3 mo) 19.7 ± 2.2; MD −1.72 (*p* = .001) (<23 threshold for impairment) *Several subscales of the MMSE were found significant at 3-month follow-up (attention and calculation, recall & action performance, MD range −0.79 to −0.33), all other domains were found to be non-significant (orientation, information encoding, & lingual skills, MD range −0.15 to −0.03). **Depressive symptoms**: IG (baseline) 8.6 ± 1.7 vs. IG (3 mo) 8.2 ± 1.17(<6 pathological symptoms) **Anxiety symptoms**: IG (baseline) 13.7 ± 2.448 vs. IG (3 mo) 12.622 ± 2.292; MD 1.155 (*p* = .001) (<6 = pathological symptoms) **Functional incapacity**(each <6 pathological symptoms) Physical symptoms: IG (baseline)12.66 ± 1.34 vs. IG (3 mo) 11.63 ± 2.31; MD 1.028 (*p* = .001) Social performance: IG (baseline) 15.05 ± 2.46 vs. IG (3 mo) 14.68 ± 2.41; MD 0.37 (*p* = .001)	Very uncertain about effects (Very low^a,c,d^)
**Benzakour 2021**; Switzerland Prospective uncontrolled cohort (pre-post)	**Screening and treatment for psychiatric symptoms** (*n* = 109; 64 follow-up)	Hospitalized 16.5% ICU (in *n* = 64 with CoviCare follow-up data) NR	**PTSD**: T0: 15 (14.6%) vs. T1: 7 (10.6%) **Depressive symptoms**: T0: 20 (18.5%) vs. T1; 6 (10.0%) **Anxiety symptoms**: T0: 17 (15.7%) vs. T1 6 (10.0%)	Very uncertain about effects (Very low^a,c,d^)
**Li 2021**, China Retrospective controlled cohort	**Chinese medicine**for 28 days after discharge; 2 different oral formula twice daily, depending on type of syndrome (pathogen residue or qi and yin deficiency) (*n* = 64) vs. Western medicine or no medicines (*n* = 32)	Hospitalized All severities (22% severe or critical) Y	**Fatigue Dyspnea Insomnia Pain**: Chest tightness No significant difference in the improvement rates of symptoms, including fatigue, between the two groups (*p* > .05)	Very uncertain about effects (Very low^a,b,c,d^)
**Martin 2021**, Belgium Prospective controlled cohort	**Telerehabilitation programme**videoconferencing; supervised by a physiotherapist; home–based individual and group 50-min endurance and strength exercises 2/week for 6 week with 2–3/week unsupervised (*n* = 14) vs. patients refusing programme (*n* = 13)	Hospitalized Severe or critical illness (22% ICU)	**Dyspnea**: Sit-to-stand (< 50th %ile): IG pre 14/14 (100%) vs. post 13/14 (93%) vs. CG pre 13/13 (100%) vs. post 13/13 (100%); RR, 0.93 [0.77, 1.13] Change since baseline in dyspnea after sit-to-stand: IG 2.5 (−2–7) vs. CG 2.0 (0–6); *p* = .56 **Adverse events**: none reported	Very uncertain about effects (Very low^a,c,D^)
Early post-acute interventions with follow-up >16 weeks post-Covid 19				
**Meije 2021**, Spain Prospective uncontrolled cohort	**Outpatient assessment with referrals**(standard follow-up protocol checklist of symptoms and adverse events, medical history, physical examination, laboratory testing including chest x-ray) medical follow-up as required (50.3% patients; 27.1% to pulmonologist) (*n* = 294)	Hospitalized Variable severity (1% IV; ICU 8.9%) *N*	**At post discharge assessment vs. 7 mo follow-up**: **Proportion with persistent symptoms**: 228 (77.6%) vs. 147 (50%) **Pain**: Migraine 19 (6.5%) vs. 12 (4.1%) Chest pain 30 (10.2%) vs. 8 (2.7%) **Psychopathology**: Need for psychological medication 35 (11.9%) vs. 30 (10.2%) Fear of relapse 68 (23.1%) vs. 86 (29.3%)	Very uncertain about effects (Very low^a,c^)
			**Dyspnea**: 88 (29.9%) vs. 28 (9.5%) **Insomnia**: 62 (21.1%) vs. 54 (18.4%) **Functional disability**: 60 (20.4%) vs. 57 (19.4%)	Very uncertain about effects (Very low^a,b,c^)

Abbreviations: CG: control group; DLCO: diffusing capacity of the lungs for carbon monoxide; ICU: intensive care unit; IG: intervention group; IV: invasive ventilation; MD: mean difference; MMRC: modified Medical Research Council dyspnea scale; MMSE: mini mental state examination; mRC: modified Rankin Scale; NR: not reported; OR: odds ratio; QoL: quality of life; RR: relative risk; vs.: versus.

*Assessed using GRADE. Certainty was rated down by 0, 1, or 2 levels for risk of bias (a), indirectness in outcome (b), inconsistency/lack of consistency (c), and imprecision/ wide confidence intervals (d); capital letters indicates very serious concern. The certainty rating applies similarly to each outcome within each row.

Table 3 summarizes the findings for this question; the certainty ratings in the last column apply similarly to each outcome within the same row (i.e. we assessed the certainty of the outcomes separately but grouped findings with similar certainty in the table). With the exception of steroids and antibiotics, we identified single studies for all interventions. Across all intervention-outcome comparisons, the certainty of evidence was found to be very low, mainly due to risk of bias, inconsistency/lack of consistency (i.e. single study effect), and in some instances imprecision. Four studies (*n* = 896) [41,47,58,59] reported on steroid use during the acute phase in hospitalized patients with dyspnea assessed at 12–16 weeks post Covid-19. Two studies (*n* = 675) [19,36] reported on the use of antibiotics during the acute phase in hospitalized and home-isolated participants, with fatigue assessed at ≥6 months after Covid-19 diagnosis. The certainty of evidence for both comparisons was assessed as very low due to serious concerns about risk of bias and inconsistency.

## Discussion

These systematic reviews were conducted in response to the growing recognition of an emerging threat and anticipated burden of post Covid-19 condition. The review focuses on studies collecting data during the early variants of the pandemic and prior to emergence of variant of concern and of widespread vaccination. In this manner, the findings are not at risk for confounding based of variants or vaccination status and are highly applicable to people who have experienced post Covid-19 condition for some time.

### Risk factors

Most of the findings had low or very low certainty evidence, often due to concerns related to risk for bias and inconsistent findings across the studies (in case of a pooled estimate) or a single study effect, although generally the reported outcomes aligned well with the review question (i.e. no indirectness). The only risk factors found to have a moderate certainty in their association with more than one post Covid-19 condition outcome were female sex and Covid-19 illness severity during the acute phase. Based on this evidence, being a female is probably (moderate certainty) associated with a small-to-moderate increase in post Covid-19 non-recovery, fatigue and dyspnea, while severe/critical acute Covid-19 (versus not severe/critical) has probably a large association with cognitive impairment (among those who were hospitalized with Covid-19), small-to-moderate association with non-recovery (among mixed populations), and little-to-no association with dyspnea (among those who were hospitalized with Covid-19). Among other risk factors, hospitalization during the acute phase may (low certainty) be associated with a large increase in non-recovery, dyspnea, and reduced return to work. We did not identify a large association for any other risk factor-outcome comparisons.

These findings add considerably to the existing evidence. A recent scientific report by the Belgian Healthcare Knowledge centre identified female sex and level of care received during acute Covid-19 as potential risk factors for long-term outcomes following Covid-19 [60]. The report was limited to studies conducted in Europe and the USA with symptoms reported at ≥4 weeks after the disease onset. Our review additionally involves a formal data synthesis and assessment of evidence certainty which were not included in the Belgian report. Findings from a recent rapid review by National Institute for Health and Care Excellence (NICE) also indicate that female sex and severity of acute Covid-19 illness (i.e. hospitalization, ICU admission) increase the risk of developing persistent symptoms after initial Covid-19 infection [30]. Our certainty for these findings was higher, possibly because we restricted inclusion to higher quality studies (having adjustment) and followed more rigorous methods for study selection and data synthesis. Our findings are also supported by a rapid review of risk factors associated with chronic Covid-19 symptoms, undertaken by Alberta Health Services (AHS) [61]. In their review, among multiple risk factors identified as having a significant association with post Covid-19 condition were female sex in previously hospitalized Covid-19 patients, and hospitalization and ICU admission in non-hospitalized and hospitalized populations, respectively. The findings in the AHS rapid review were based on statistical significance, while in our review we relied on the magnitude of association and its certainty using various criteria since lack of a statistical significance should not be taken as a lack of association [62]. Compared with the AHS review, we limited outcomes to 30 days or beyond the date of Covid-19 diagnosis and employed a strict population size criterion for eligibility. Despite methodological variation across these reviews and timing of outcome assessment, findings support that there is probably a link between these risk factors and post Covid-19 outcomes. While the association between acute Covid-19 severity and persistent symptoms is plausible, it is yet not clear why females are at a higher risk of post Covid-19 condition. Our previous review on risk factors for Covid-19 illness severity, also relying on adjusted findings, found moderate certainty evidence for little-to-no difference between females and males so the finding may not be related to the severity of the initial illness [26]. All of the studies in this review that reported on sex adjusted their analysis for age and at least one marker of acute Covid-19 severity, and findings did not significantly differ when comparing different settings (hospitalized vs. non-hospitalized vs. mixed). Further, acute illness severity but not sex had a large association with cognitive impairment.

The certainty of evidence for individual pre-existing conditions was low or very low, and most of the evidence was for little-to-no association. Although existing evidence indicates older age might increase the risk of post Covid-19 symptoms regardless of the follow-up length [30,61,63,64], most of our evidence was low certainty for little-to-no association apart from more functional incapacity in ≥60 versus <60-year-old hospitalized patients. Our findings may differ from other reviews due to our strict eligibility criteria as we only included studies reporting outcomes at least 12 weeks after disease onset, to minimize the likelihood of symptoms associated with acute Covid-19, with at least 300 participants, and controlling for a minimum set of confounders (i.e. 2 or more of age, sex, comorbidities, and illness severity). Also, we excluded studies where all participants were admitted to an ICU to avoid overlapping of post Covid-19 condition with post-intensive care syndrome, which is characterized by similar features to post Covid-19 [65].

#### Limitation of the evidence and future direction

Our findings are mostly applicable to longer-term consequences of Covid-19 occurring ≥22 weeks after diagnosis or illness onset as we identified only a few studies with shorter follow-up length. Also, despite the large volume of data emerging, many studies came from a hospitalized population. Of all the included studies, we identified only one that was exclusively in children who were previously hospitalized with Covid-19 [40]. The study reported on several potential risk factors including age, sex, acute Covid-19 severity, obesity, and allergic diseases, however, none were identified as having a strong association with outcomes of our review. Evidence was also sparse in relation to pre-existing socioeconomic variables (e.g. race/ethnicity, income, education, employment) and marginalized groups including indigenous communities, institutionalized populations, and persons with disability, despite being listed as priority populations for Covid-19 policies by several jurisdictions. Moreover, current studies are mostly based on self-reported outcome and exposure data, which could be subject to recall bias and misclassification, and may limit generalizability of the evidence to other populations [30]. There is also the possibility of over/under-estimation of the reported associations as people from certain populations may be under-represented in primary studies [66]. Further, based on the current literature, it is still challenging to determine if persistent symptoms are actually attributed to initial Covid-19 or would have occurred independent of the infection [60]. To overcome these limitations, more robust evidence of risk factors is required particularly in non-hospitalized populations and community settings. Use of administrative data and establishing universal outcome definitions and assessment methods could ensure robustness of the evidence.

### Interventions

To the best of our knowledge, this is the only systematic review to explore interventions that could potentially prevent post Covid-19 outcomes evaluated at least 12 weeks after the disease onset. Based on the current evidence, we are very uncertain about the effect of any intervention to prevent persistent symptoms associated with Covid-19. Most of the evidence came from single studies in hospitalized patients with wide variation in sample size and methodology. Interventions reported in multiple studies (steroids and antibiotics) had either inconsistent findings or did not report on similar outcomes relevant to our review question. With the exception of the one pilot trial [56], all identified studies were non-randomized and all had concerns about risk of bias due to multiple issues.

There is some evidence suggesting that rehabilitation might have a beneficial effect on recovery from Covid-19 [67–70], and that there may be a positive effect of medications on post-acute clinical outcomes of Covid-19 [71,72]. These studies were not included in our synthesis due to either short follow-up time or the outcomes not being prioritized by the working group and patient panel for inclusion. Regarding Covid-19 vaccination as a possible preventive intervention, findings from a recent (published since our search) observational study indicate that receiving Covid-19 vaccine after getting infected might offer some protection against long Covid-19 outcomes [73]. Other studies we identified from our search or have identified thereafter did not meet inclusion criteria (e.g. vaccination before infection [74,75], indeterminant timing of vaccination [76], entire sample had post Covid-19 condition [77,78]). Further updates of this review will likely identify more evidence about the possible effects of vaccination. Variations in definition of post Covid-19 outcomes and timing of assessment make it difficult to draw conclusions or compare the findings of our review with other studies. Additionally, a lack of well-designed randomized or quasi-randomized trials significantly limits our understanding of possible effects of any potentially preventive interventions. The living systematic review by a Cochrane group shows that the focus of scientific research is shifting from treatment of acute Covid-19 to post-acute and chronic phases of Covid-19 [67]. Thus, continuous review and assessment of the rapidly emerging evidence is important to better shape our understanding as the body of evidence grows.

### Strengths and limitations of our review

We followed established guidelines for systematic reviews to provide methodologically rigorous syntheses of the available evidence on risk factors associated with post Covid-19 conditions and potentially preventive interventions. The review questions and outcomes were informed by input from clinical experts, stakeholders, and a patient panel. A wide range of risk factors and preventive interventions were considered in our reviews. Our findings are based on our certainty about associations/magnitudes of effect reaching predefined thresholds, which is informed by several factors, rather than reliance of statistical significance that is commonly used across the literature.

Despite these strengths, there are some limitations in our review. As with other reviews, there is a possibility of missing relevant studies by our search, although this was mitigated through searching grey literature resources and references of the included studies and relevant reviews. Involving experienced reviewers and selecting studies in duplicate further reduced the possibility of any important studies being missed. Though our searches were conducted in 2021 the applicability is high to people having Covid-19 in the early phases of the pandemic or without potential protection by vaccination. Our search was limited to English or French studies and this might have resulted in missing studies from jurisdictions where other languages are commonly used for publication. Further, although we followed the standard guidelines to select thresholds for magnitude of association, the findings might change if different thresholds were selected. Our findings are limited to data using the currently accepted definition of post Covid-19 condition, with persistent symptoms at ≥12 weeks, and may not be fully applicable to other definitions should this change once more evidence is known about the longer term impacts of the condition.

## Conclusion

Being a female is probably (moderate certainty) associated with a small-to-moderate increase in post Covid-19 non-recovery, fatigue and dyspnea, and having a severe or critical acute Covid-19 illness (versus not severe/critical) probably has a large (2-fold or more) association with increased cognitive impairment, a small-to-moderate association with more non-recovery, and little-to-no association with dyspnea. Though with low certainty, hospitalization during the acute phase may have large associations with more non-recovery and dyspnea and less return to work. All other evidence on risk factors was low certainty for small-to-moderate or little-to association or very low certainty. Evidence on possible preventive interventions was mostly in hospitalized patients and observational in nature, and all provided very low certainty evidence. Continuous assessment of the emerging evidence is important to better shape our understanding of this condition; determining whether different variants and vaccination impact outcomes will be important. Sufficiently powered prospective trials of preventive interventions are warranted.

### Policy implications

Post Covid-19 condition is becoming a public health challenge for many communities and governments. To be effective and efficient, public health policies and programmes that will be rolled out in response to this challenge need careful evaluation and assessment of available resources. Synthesizing high-quality evidence to identify risk factors of persistent Covid-19 symptoms and possible preventive interventions is therefore important in highlighting areas where policy-makers can take action to mitigate the longer term outcomes of this pandemic. The wide range of post Covid-19 condition outcomes and associated risk factors emphasize the need for a coordinated multidisciplinary approach to assessment and management strategies. We found that female sex and severe or critical acute-phase Covid-19 are independent markers for greater risk of developing post-Covid complications. These findings imply that public health guidelines in relation to surveillance, screening services, and other services such as, access to sickness and disability benefits, might need to prioritize these groups. Interventions targeting fatigue, dyspnea, and cognitive impairment (especially in those with previous severe illness) may be good to prioritize for development and evaluation and to provide evidence on their effects. Further, inputs from patients and primary care providers should be taken into account when developing new care pathways and appropriate services, including long-term follow-up, rehabilitation and support groups, to ensure management and treatment strategies are tailored to patient needs and the disease manifestations.

## Supplementary Material

Supplemental Material

## Data Availability

All of the data extracted for this review are included in the manuscript and associated supplementary files.
